# Multiparametric MRI in the PSA Screening Era

**DOI:** 10.1155/2014/465816

**Published:** 2014-08-27

**Authors:** Arvin K. George, Peter A. Pinto, Soroush Rais-Bahrami

**Affiliations:** National Cancer Institute, Urologic Oncology Branch, National Institutes of Health, 10 Center Drive, 2950-W, Building 10, CRC Room 2W-5940, Bethesda, MD 20892-1210, USA

## Abstract

Prostate cancer remains significant public health concern amid growing controversies regarding prostate specific antigen (PSA) based screening. The utility of PSA has been brought into question, and alternative measures are investigated to remedy the overdetection of indolent disease and safeguard patients from the potential harms resulting from an elevated PSA. Multiparametric MRI of the prostate has shown promise in identifying patients at risk for clinically significant disease but its role within the current diagnostic and treatment paradigm remains in question. The current review focuses on recent applications of MRI in this pathway.

## 1. Introduction

An estimated 233,000 newly diagnosed cases of prostate cancer (CaP) are estimated for 2014, with a projected 29,480 CaP deaths in the same year [1]. The 2008 US Preventive Services Task Force (USPSTF) recommendation against prostate cancer screening in patients >75 years has begun to reveal a reversal in stage migration with a decreased incidence of localized disease and an increase of distant disease in this age group [2]. The most recent USPSTF reevaluation for prostate specific antigen (PSA) screening put forth in 2012 resulted in a grade D recommendation [3]. The ultimate impact has yet to be determined but may yield a similar fate if widely adopted. The debate surrounding population-based PSA screening follows conflicting level 1 evidence with the determination based ultimately on an unfavorable harms/benefits ratio for screening. Despite overdetection (of clinically insignificant CaP), the fact remains that underdiagnosis (of clinically significant prostate cancer) persists with a third of patients pathologically upgraded from initial biopsy to radical prostatectomy [4]. Though PSA is an imperfect tool, once a patient has elected to undergo screening, the decision for biopsy is made based on this value with no absolute threshold to move forward to biopsy. No PSA value exists that completely excludes high grade disease, and the trend to lowering PSA cutoffs coupled with increased sampling has resulted in a growing number of biopsies, driven overdetection, and exposed patients to risks associated with diagnosis and treatment of potentially indolent disease.

The shortcomings of PSA and prostate biopsy have propelled the search for alternative measures to improve its diagnostic yield and efficiency. Transrectal ultrasound (TRUS) guided biopsy of the prostate currently remains the gold standard for tissue diagnosis but itself has limitations. The systematic yet random and blinded sampling of TRUS biopsy served a useful purpose from its introduction in the 1980s, when biopsy of palpable, large-volume disease was representative of whole gland pathology. The introduction of PSA and subsequent identification of organ-confined, low volume disease have made TRUS biopsy gradually more antiquated and now CaP remains the only solid organ tumor diagnosed without tumor imaging and a directed sampling method.

**Figure 1:**
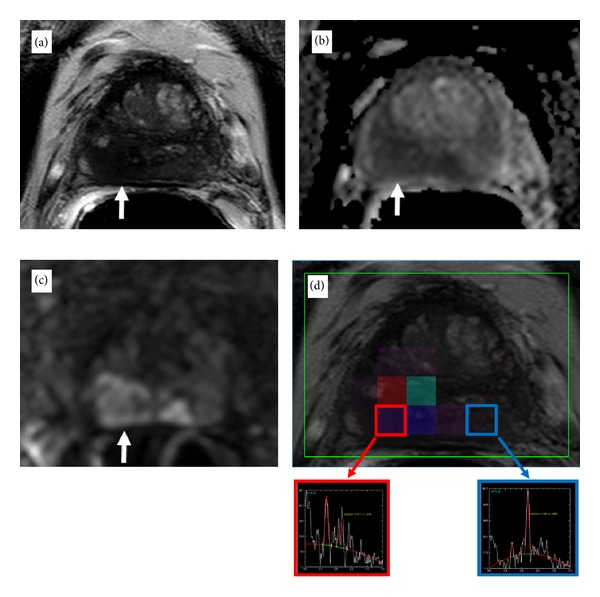
(a) Axial T2-weighted imaging, (b) ADC map from DW MRI, (c) DCE MRI demonstrating early and avid gadolinium enhancement, and (d) spectroscopic imaging depict a right mid-base peripheral zone lesion (arrow).

*Multiparametric MRI (mp-MRI) of the Prostate.* The role of prostate biopsy has evolved from a purely diagnostic tool to include one that informs clinical decision-making. As such, it is essential to ensure that the appropriate individuals receive a biopsy thus averting unnecessary harm and that biopsy information represents what it truly intends to measure. In an effort to improve on the current standard, PSA-derived markers, PSA kinetics, and patient characteristics including genomic profiling and imaging have all been investigated to refine patient selection with variable results. The application of mp-MRI of the prostate has emerged as a powerful tool that provides detailed anatomical and functional information where it is currently lacking. In addition to high resolution T2 weighted (T2W) (anatomical) imaging, the integration of diffusion weighted imaging (DWI), dynamic contrast enhanced (DCE) imaging, and spectroscopic (functional) imaging in combination has allowed radiologists to better identify areas of benign and potentially malignant disease (Figure 1) [5]. The large amount of information obtained by imaging enables its use as a complimentary tool to PSA at the time of biopsy to direct sampling, before treatment for staging and after therapy for monitoring disease and recurrence. A growing body of literature supports the advantages of mp-MRI but a fundamental challenge has been understanding the most appropriate role in the current diagnostic and treatment paradigm. The goal of this review is to summarize the current indications and applications of mp-MRI of the prostate in the era of PSA screening.

## 2. Integration of MP-MRI

### 2.1. Ab Ovo

The use of mp-MRI as a screening tool has recently been compared to PSA and digital rectal exam [6]. A mp-MRI consisting of T2W and DWI series was proposed as a limited diagnostic study that can be completed in 15 minutes and avoid additional time and cost associated with DCE and spectroscopy. It was found in a biopsy-naïve cohort that the area under the curve (AUC) for prostate cancer detection was 0.66 for PSA alone and 0.80 for MRI when >1 lesion was identified. The effect was cumulative when PSA density (PSAD) and MRI were taken together with an AUC of 0.87. A positive MRI had a sensitivity of 90% but did suffer from low specificity (54%). By combining PSA, PSAD, and MRI, these improved with corresponding increases in the negative and positive predictive values, specifically for Gleason ≥7 disease. Though encouraging, these results preliminary require further investigation for MRI as a “screening” modality or rather an additional filter prior to biopsy and in its current state serve as useful adjunct to PSA testing.

### 2.2. Before Biopsy

An elevated PSA will prompt a TRUS biopsy modelled towards sampling areas where cancer is most “likely” to be found—the peripheral zone. The resolution of TRUS is generally insufficient to identify areas suspicious for tumor and biopsy proceeds in a random fashion with the goal of obtaining results that will reflect whole gland pathology. Indiscriminate biopsy systematically undersamples areas not within the standard biopsy zone including the anterior peripheral zone and transition zone where up to 25% of prostate cancers may lie [7, 8]. Prebiopsy imaging can be effectively utilized as a triage tool to identify patients at risk and provide targets for biopsy [9]. Villers et al. demonstrated that, on whole mount histology at radical prostatectomy, there is no evidence of clinically significant prostate cancer in 95% of the areas where mp-MRI failed to identify a focus of concern [10]. Additionally, the utility of mp-MRI has been evaluated across established PSA cutoffs where the greatest benefit was seen in patients with a PSA ≥ 4. Specifically the use of mp-MRI and fusion biopsy in patients with a PSA ≥ 5.2 captured 90% of upgrading from standard 12-core to targeted biopsy [11].

An extended sextant 12-core TRUS biopsy will detect cancer in about a quarter of patients [12]. Sampling error in large areas of the peripheral zone, in addition to undersampling of alternate locations, gives rise to the high false negative rate and corresponding poor negative predictive value (NPV) of random biopsy. Lesions suspicious for cancer on MRI provide targets for image-directed biopsy that can be missed with a conventional systematic biopsy. Lesions within the anterior prostate and lower apical region can be readily identified and sampled of which 55% reveal cancer with targeted biopsy, having specific implications to treatment where positive margins are more prevalent [13]. MRI has provided additional value where PSA has fallen short. Though sensitive, PSA has limited ability to discriminate between causes for elevation. Though significant overlap has restricted its use, median apparent diffusion coefficient (ADC) values of prostatitis were found to be significantly higher when compared to cancer foci [14]. Additionally, MRI and fusion biopsy are able to control for relative undersampling related to larger prostate volumes while maintaining cancer detection rates of 48% in prostates >40 cc and without overlooking high risk disease [15].

The strongest support for the use of mp-MRI has been in those patients with negative TRUS biopsy and persistently elevated PSA. When MP-MRI targets were used to guide biopsy, cancer was found in 37–59% of cases [16–18]. The NPV of MP-MRI for clinically significant cancer ranged from 79 to 95% when a transperineal mapping biopsy was used as the reference standard in patients with prior negative biopsies, suggesting that MP-MRI may be an important adjunct in ruling out disease in this challenging patient demographic [19].

### 2.3. During Biopsy

Targeted biopsy via an in-bore, cognitive fusion, or software-based fusion platform has allowed for directed biopsy of a specific lesion identified on prebiopsy imaging. Siddiqui et al. demonstrated cancer detection rates of up to 54% with a third of patients being upgraded from random cores to targets [20]. Targeted biopsy detected up to two thirds more patients with clinically significant disease than random biopsy and detected one-third of less clinically insignificant disease. The upgrading and detection of clinically significant CaP have been validated with independent series [21]. A recent systematic review confirmed that, on a per core or a per patient basis, targeted biopsy is able to detect clinically significant CaP with fewer biopsies with a reduction in detection of insignificant disease [22].

### 2.4. Before Definitive Therapy

The incorporation of PSA into various prognostic models has provided valuable information for pretreatment prediction of risk for organ confined disease. MP-MRI is able to identify areas of seminal vesicle invasion (SVI) (that can potentially undergo subsequent targeted biopsy for tissue confirmation), extracapsular extension (ECE), and even pelvic lymph node involvement. Recent series have reported sensitivity of >80% and specificity of >90 [23–25]. Soylu et al. reviewed 131 patients who underwent radical prostatectomy with preoperative MRI finding a 17.6% rate of seminal vesicle invasion on final pathology. The authors found that T2-weighted images yielded a high specificity and negative predictive value (93.1% and 94%, resp.), and addition of DWI was able to significantly improve specificity and positive predictive value, which remained limited (70%). Incorporation of DCE images did not identify significant improvement [26]. At the National Cancer Institute, all patients who underwent fusion-guided targeted biopsy of the seminal vesicle preoperatively were found to have concordant pathology with final histology at radical prostatectomy despite a small sample size of four patients. Preoperative tissue diagnosis can allow for accurate risk stratification and potentially modify treatment selection. Similarly, the predictive value of mp-MRI for ECE was evaluated in 183 patients undergoing prostatectomy and stratified by risk groups according to the D'Amico criteria. On multivariate analysis, only PSA and stage on mp-MRI were associated with ECE on final pathology with mp-MRI being the strongest predictor (OR 1.1 versus 10.3) [27]. Detailed anatomic imaging which provides information regarding tumor location or possible ECE can also determine surgical approach including the ability to provide a nerve sparing operation and was noted to influence surgical management in 26% of cases [28].

High-quality imaging is a fundamental component for patient selection for focal therapy of prostate cancer [29]. Until recently, the limited ability of MRI to delineate intraprostatic anatomy restricted its use for this purpose. MP-MRI provides excellent discrimination of suspicious lesions and upon tissue confirmation at targeted biopsy can provide information regarding lesion location, volume, and relation to vital structures including the urethra, neurovascular bundle, and rectum, in addition to confirmation or low or intermediate risk pathology. The use of mp-MRI is now being integrated into consensus statements defining best practice guidelines for the identification and treatment of prostate cancer with focal therapy [30].

### 2.5. After Definitive Therapy

The most valuable utilization of PSA remains after whole-gland treatment, where increases in PSA correlate well with disease recurrence. MP-MRI can be used to identify local recurrence after both radical prostatectomy and external beam radiation with functional imaging sequences driving detection in these patients [31]. The addition of targeted biopsies in cases of recurrence after radiation therapy allows for a significantly improved cancer detection rate (83% versus 20%) over that of random sampling [32]. When there is suggestion of recurrence postradical prostatectomy, PSA alone is insufficient in offering information regarding the presence of local or distant metastasis. In this setting, mp-MRI is a useful adjunct and has been shown superior to PET-CT for local recurrence and equally excellent in the detection of bony lesions [33]. In the case of focally treated lesions, PSA for posttreatment surveillance is inadequate as imaging directs therapy and should be employed in followup to ensure the absence of de novo lesions and to direct targeted biopsy. Additionally, PSA is not reflective of disease persistence or recurrence and no current stand exists in the setting of residual normal prostate or satellite nondominant cancer foci that have not been treated or identified.

### 2.6. During Treatment

The detailed anatomic information afforded from mp-MRI enables its versatile use intraoperatively where PSA falls short. Higher PSA values may provide generalized information, from which inferred risk is calculated based on large datasets but fails to provide patient-specific information that can guide surgery. Improved risk stratification, imaging, and novel therapeutic approaches have enhanced efforts for focal therapy in prostate cancer. Focal therapy techniques including high intensity focused ultrasound and laser induced thermal therapy are largely guided by real-time MRI. MRI can assist in tumor localization and probe placement in addition to treatment monitoring intraoperatively. Novel MR-TRUS fusion platforms for guidance of focal therapy are also beginning to emerge [34]. MR Thermometry can provide critical information on margins, ensuring a tight ablation zone and lethal dose of energy to the target area [35].

### 2.7. Active Surveillance

Overdetection of clinically insignificant, low-grade, low-volume CaP has spurred the adoption of active surveillance (AS) as an accepted management strategy. AS allows patients with low risk disease to defer definitive therapy, potentially indefinitely, until objective evidence of disease progression is noted. PSA is utilized as both an entry criterion and a mainstay of surveillance in conjunction with random prostate biopsy for AS. MP-MRI and targeted biopsy are able to better differentiate those patients with indolent disease, selecting patients who better represent low-risk disease [36]. Of patients entering an active surveillance protocol based on Johns Hopkins criteria, 29% of patients are no longer candidates for AS after targeted biopsy. A nomogram derived from MRI parameters has been developed to determine eligibility for AS but requires independent validation [37]. Additionally, the stability or progression of MRI findings is currently being investigated to establish its correlation with pathologic changes. Patients with small lesions on surveillance demonstrate relative stability on imaging findings and pathology within 2 years suggesting the indolent nature of low volume findings, and support a longer interval follow-up [38]. It should be noted however that progression of MR spectroscopic findings have recently provided with objective evidence for progression from low-risk to high-risk disease on targeted biopsy  [39]. MRI with targeted biopsy allow for accurate re-sampling of prior areas of concern. The location of prior positive cores can be revisited directly by assigning these areas as targets based on localization from the prior biopsy allowing for direct evaluation of potential progression by volume or grade [40]. In the future, MP-MRI may augment the use of PSA and DRE and allow for targeted biopsies to resample areas of known disease.

### 2.8. Future Directions

Investigation of novel technologies harnessing the properties of mp-MRI is showing promise in the staging of prostate cancer. Current models including the UCSF-CAPRA scoring system and the Partin tables integrate PSA to provide valuable insight into presence of metastasis and predictions of organ confined disease. Ultrasmall superparamagnetic particles of iron oxide (USPIO) have shown promise in the preoperative staging of bladder and prostate cancer patients, allowing for detection of metastasis. DWI combined with USPIO has demonstrated potential in discrimination of malignant versus benign pathology in even normal size lymph nodes, though wide adoption has been hindered due to variability in interpretation [41]. In addition, DWI MRI in conjunction with newer contrast agents such as gadofosveset trisodium is being used to evaluate lymph node staging in clinical trials for rectal cancer and its role in prostate cancer is currently being studied [42].

Finally, Positron emission tomography (PET) by itself demonstrates high sensitivity for prostate cancer detection but nonspecific uptake in benign lesions limits its diagnostic utility. However, PET/MRI has recently shown promise with a sensitivity and specificity for CaP detection in lesions >5 mm being 84% and 80%, respectively, when correlated with whole mount pathology at radical prostatectomy [43]. Furthermore, semiquantitative [18F]fluoroethylcholine uptake in identified lesions is able to discriminate between Gleason >6 CaP with a specificity of 90% and a positive predictive value of 83%, which is an improvement over that of prostate biopsy results when compared to whole mount specimens.

## 3. Limitations

The novel applications of MP-MRI are encouraging and but are not a panacea to our PSA woes. MRI interpretation is challenging, requiring considerable experience before proficiency. As mp-MRI has gained acceptance, the need for standardized protocols and reporting and high quality studies has been realized [44]. Determining cost efficacy will be essential before its use becomes ubiquitous, but undoubtedly as use increases costs will decline.

## 4. Conclusion

PSA remains the cornerstone to screening, staging, and surveillance after treatment despite its numerous shortcomings. Currently, mp-MRI is proving an essential adjunct to supplement PSA in proper patient selection for biopsy, treatment, and surveillance. High-quality studies are required to validate the initial encouraging body of literature supporting the use of MRI in this arena.
